# ONLINE COGNITIVE MONITORING IN A SIMULATED ANTI-AMYLOID TRIAL: ADHERENCE AND COGNITIVE CHANGE

**DOI:** 10.1093/geroni/igad104.2755

**Published:** 2023-12-21

**Authors:** Jeffrey Kaye, Nora Mattek, Jennifer Marcoe, Amanda Mar, Yanan Shang, Aimee Pierce, Kevin Duff, Adriana Hughes

**Affiliations:** Oregon Health & Science University, Portland, Oregon, United States; Oregon Health & Science University, Portland, Oregon, United States; Oregon Health & Science University, Portland, Oregon, United States; Oregon Health & Science University, Portland, Oregon, United States; Oregon Health & Science University, Portland, Oregon, United States; Oregon Health & Science University, Portland, Oregon, United States; Oregon Health & Science University, Portland, Oregon, United States; University of Minnesota, Minneapolis, Minnesota, United States

## Abstract

Remote assessment of cognitive function is increasingly of interest for monitoring cognitive decline. Additional advantages of remote monitoring are more frequent assessments and the examination of change over time, which may yield more sensitive indicators of cognitive status. This concept was tested in DETECT-AD (Digital Evaluations and Technologies Enabling Clinical Translation for Alzheimer’s Disease), an ongoing simulated anti-amyloid trial using digital biomarkers as outcome measures. Adherence to testing, relationships to age, global cognition, and SUVR, and trajectories of change were analyzed in the SMART (Survey for Memory, Attention and Reaction Time) test, a brief measure of visual-grid memory and executive function (Trails A/B, Stroop Color-Word), self-administered online every four weeks. To date (02/28/2023), 46/100 have been enrolled with mean(SD): follow-up=31.0(7.9) weeks (no dropout); age=78(6.6); 63% women; MoCABaseline=25.5(2.3); PET SUVR=1.09 (0.20; range=0.850-2.07). SMART surveys (n=184) completed online used a desktop (26%), laptop (48%), tablet (20%), or smartphone (7%). A mean of 4(2.0; range=1-8) SMART surveys/participant were completed with 96% completed tests. Older age significantly correlated with longer overall completion times (p=0.02), Trails-B (p< 0.01) and Stroop (p=0.02) times, and more click counts on Trails-B (p=0.03). MoCA total score significantly correlated with shorter completion times on overall SMART (p=0.02), Trails-A (p< 0.01), and Trails-B (p=0.03). There were no relationships between SUVR and SMART. Total SMART completion time decreased over multiple administrations (p=0.03), driven by shorter Trails-B time (p=0.01). Monthly online screening of cognitive function in a clinical trial setting is well maintained yielding novel information over time.

